# Identification and Characterizations of Novel, Selective Histone Methyltransferase SET7 Inhibitors by Scaffold Hopping- and 2D-Molecular Fingerprint-Based Similarity Search

**DOI:** 10.3390/molecules23030567

**Published:** 2018-03-02

**Authors:** Hong Ding, Wen Chao Lu, Jun Chi Hu, Yu-Chih Liu, Chen Hua Zhang, Fu Lin Lian, Nai Xia Zhang, Fan Wang Meng, Cheng Luo, Kai Xian Chen

**Affiliations:** 1 School of Pharmacy, Shanghai University of Traditional Chinese Medicine, 1200 Cailun Road, Shanghai 201203, China; hding@simm.ac.cn; 2 CAS Key Laboratory of Receptor Research, State Key Laboratory of Drug Research, Shanghai Institute of Materia Medica, Chinese Academy of Sciences, 555 Zuchongzhi Road, Shanghai 201203, China; wenchaolu@simm.ac.cn (W.C.L.); hujunchi@simm.ac.cn (J.C.H.); fulinlian1986@simm.ac.cn (F.L.L.); nxzhang@simm.ac.cn (N.X.Z.); e-cluo@simm.ac.cn (C.L.); 3 Shanghai ChemPartner Co., Ltd., #5 Building, 998 Halei Road, Shanghai 201203, China; yzhliu@chempartner.com (Y.-C.L.); chhzhang@chempartner.com (C.H.Z.); 4 Department of Chemistry and Chemical Biology, McMaster University, 1280 Main Street West, Hamilton, ON L8S 4L8, Canada

**Keywords:** SET7, inhibitor, similarity search, ligand-based drug design, chemical biology probe

## Abstract

SET7, serving as the only histone methyltransferase that monomethylates ‘Lys-4’ of histone H3, has been proved to function as a key regulator in diverse biological processes, such as cell proliferation, transcriptional network regulation in embryonic stem cell, cell cycle control, protein stability, heart morphogenesis and development. What′s more, SET7 is involved inthe pathogenesis of alopecia aerate, breast cancer, tumor and cancer progression, atherosclerosis in human carotid plaques, chronic renal diseases, diabetes, obesity, ovarian cancer, prostate cancer, hepatocellular carcinoma, and pulmonary fibrosis. Therefore, there is urgent need to develop novel SET7 inhibitors. In this paper, based on DC-S239 which has been previously reported in our group, we employed scaffold hopping- and 2D fingerprint-based similarity searches and identified DC-S285 as the new hit compound targeting SET7 (IC_50_ = 9.3 μM). Both radioactive tracing and NMR experiments validated the interactions between DC-S285 and SET7 followed by the second-round similarity search leading to the identification ofDC-S303 with the IC_50_ value of 1.1 μM. In cellular level, DC-S285 retarded tumor cell proliferation and showed selectivity against MCF7 (IC_50_ = 21.4 μM), Jurkat (IC_50_ = 2.2 μM), THP1 (IC_50_ = 3.5 μM), U937 (IC_50_ = 3.9 μM) cell lines. Docking calculations suggested that DC-S303 share similar binding mode with the parent compoundDC-S239. What′s more, it presented good selectivity against other epigenetic targets, including SETD1B, SETD8, G9a, SMYD2 and EZH2. DC-S303 can serve as a drug-like scaffold which may need further optimization for drug development, and can be used as chemical probe to help the community to better understand the SET7 biology.

## 1. Introduction

In the epigenetic landscape, histone methyltransferases (HMTs) play an essential role in various biological processes including cell cycle progression [1], cell differentiation [2], development [3] as well as other biological processes [4]. Besides, HMTs are involved with the pathogenesis of cancers [5,6,7,8], immune-mediated diseases [9], thus they have been the hot targets for cancer therapy in both academia and industry. HMTs can be categorized into two groups based on structural features: (i) SET domain-containing subfamily, such as SET7 (SET domain-containing lysine methyltransferase 7, also called SETD7, SET9, KMT7), EZH2 (Enhancer of Zest Homologue 2) and SUV39H1 and (ii) non-SET domain containing subfamily, such as DOT1-L (Disruptor of Telomeric silencing 1-Like) [10].

SET7 is the only epigenetic member that specifically monomethylates ‘Lys-4’ of histone H3 and emerging evidences have proved SET7′s unique role in transcriptional regulations [11] (Table 1), DNA repair [12], cell cycle control [12,13,14]. Due to its catalytic activity on diverse non-histone substrates, SET7 also displays a special role in a lot of biological processes (Table 1) and is involved in cell proliferation [15,16], transcriptional network regulation in embryonic stem cell [17,18], cell cycle control [19], protein stability [20,21,22,23], heart morphogenesis and development [24], as well as other biological functions. Notably, its role in regulating p53, whose mutant isoform is an important cancer therapy target [25], remains controversial [26].

In addition, SET7 is reported to be a key regulator in the pathogenies of several diseases in cluding alopecia areata [69], breast cancer [37,70], tumour and cancer progression [71,72,73,74,75,76], atherosclerosis in human carotid plaques [77], chronic renal diseases, diabetes [45,78,79,80,81,82,83,84], obesity [85], ovarian cancer [86], prostate cancer [87], hepatocellular carcinoma [71], and pulmonary fibrosis [57]. Of interest, the role of SET7 in viral infection is being uncovered. Studies revealed that SET7 can facilitate HCV replication in an enzymatic activity-dependent manner through attenuation of the IFN/JAK/STAT pathway [88]. SET7 binds with HIV-1 TAR RNA and monomethylates the HIV viral trans-activator Tat protein at Lys-51 and Lys-71, to enhance HIV transcription [63,89]. Knockdown analysis also proved that SET7 only suppresses Tat transactivation of the viral promoter and has on influence on transcriptional activity of methylation-deficient Tat Shan’s study suggested that SET7 can negatively regulate the anti-viral activity toward vesicular stomatitis virus (VSV) and influenza A by methylation at Lys-88 of virus (IAV) infection of Interferon-induced Transmembrane Protein 3 (IFITM3) [44]. What′s more, SET7 may also have a potential role in HPV (human papillomaviruses) viral survival [90]. Therefore, SET7 may be a promising target for viral infection and potent and selective inhibitor can serve as a useful chemical biology tool to elucidate the viral infection mechanism. The abberant expression patterns and dysfunction of SET7 has also been widely observed in the onset and progression of cancers. In peripheral blood mononuclear cells of patients, the histone modification patterns were greatly altered and the expression of SET7 was elevated [69]. Emerging evidence has also demonstrated its role in solid tumors. Zhang et al. demonstrated that SET7 interacts with transcription factor GATA1 and promotes downstream VEGF transcription and tumor angiogenesis [40]. Inhibition of SET7 activity by the SET7 inhibitor cyproheptadine reduced the estrogen receptor alpha expression in MCF7 cells that is important for cancer progression, phencopying the SET7 knockdown studies [37]. With SET7′s emerging role of therapy targets for cancers, diabetes, hepatocellular carcinoma, alopecia areata, pulmonary fibrosis and viral infections, a potent and selective SET7 inhibitor is in great need to serve as a chemical probe to investigate its delicate biological function and mechanism.

So far, several successful attempts have been made to develop SET7 inhibitors. (*R*)-PFI-2 [91], as resolved in the crystal complex structure, is bound to the peptide binding pocket with a high affinity (IC_50_ = 2.0 nM). Using a drug repositioning strategy, Cyproheptadine, a clinically approved antiallergy drug was reported to bind with SET7 peptide binding site as revealed in structural biology studies (IC_50_ = 1.0 μM) [61].

Computational tools and methods have been widely used for epigenetic inhibitor design and discovery and many successful cases have been reported [92]. Some epigenetic inhibitors have been identified by computational methods targeting PRMT1, DNMT1, DOTL and the protein-protein interfaces like menin-mixed lineage leukemia 1 and EZH2-EED [92]. In our previous study, two selective SET7 inhibitors DC-238 and DC-S239 were identified with an integrated virtual screening method combing pharmacophore and docking as well as chemical modifications [92].

In this paper, a new SET7 inhibitor DC-S285 was identified by scaffold hopping and 2D fingerprint -based similarity search based on the previously identified SET7 inhibitor DC-S239, which was further validated by radioactivity assay and NMR-spectroscopy methods. In cellular level, DC-S285 also inhibited cancer cell proliferation in leukemic cells and breast cancer cells. Therefore, a similarity search was performed in search of more potent inhibitors as well as for its structure-activity relationship (SAR) studies. The predicted binding mode was also investigated in accordance with its SAR. In summary, a novel and selective compound DC-S336 (IC_50_ = 1.1 μM) was identified by similarity based search.

## 2. Results and Discussions

### 2.1. Scaffold Hopping- and Similarity-Based Virtual Screening

**Figure 1:**
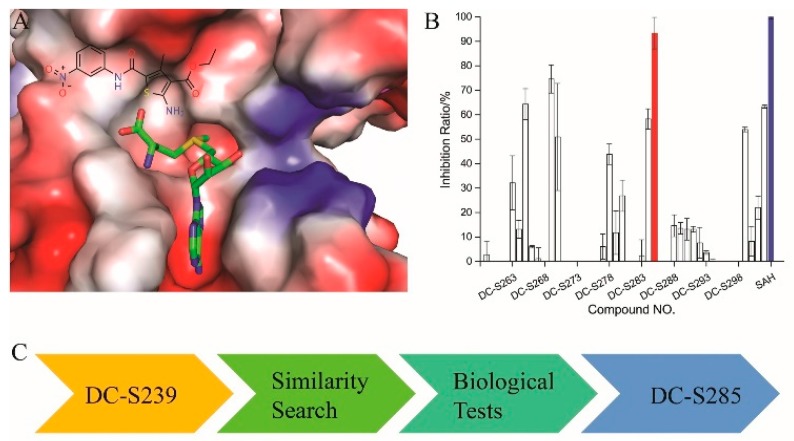
The flowchart of the combinatorial scaffold hopping and 2D fingerprint based similarity search strategies. (**A**) Chemical structure of DC-S239 and its predicted binding mode. DC-S239 is shown in sticks and the SET7 protein is shown in electrostatics surface; (**B**) Inhibition ratio of the compounds in first-round similarity search. DC-S285 is shown in red while the reference compound SAH is depicted in blue; (**C**) Workflow chart in the study.

**Figure 2:**
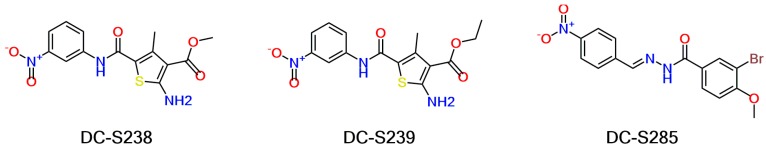
Chemical structures of DC-S238, DC-S239 and DC-S285.

Based on our previous research, two selective SET7 inhibitors, namely DC-S238 (IC50 = 4.9 μM) and DC-S239 (IC50 = 4.6 μM) were obtained (Figure 1A). Herein, with DC-S239 serving as the starting point, combinatorial scaffold hopping [93] and 2D fingerprint based similarity search strategies were employed in order to discover more SET7 inhibitors with novel chemotypes (Figure 2). The overall computational pipeline has been demonstrated (Figure 1C).

Before performing similarity searches, pre-processing of the Specs molecular database was conducted with Pipeline Pilot, version 7.5 (Pipeline Pilot; Accelrys Software Inc., San Diego, CA, USA). Because a lot of clinical trial failures and unnecessary attritions were due to poor oral bioavailability [94,95], it becomes crucial to estimate druglikeness properties at the early drug design and development stage, and therefore the Specss database was filtered by Rule of Five [96]. Recently, a series of promiscuous, assay-duping molecules, namely ‘pan-assay interference compounds’ (PAINS), were reported due to metal chelation, chemical aggregation, redox activity, compound fluorescence, cysteine oxidation or promiscuous binding against the targets, suggesting that we should get rid of those structures in early state [97,98,99,100]. Therefore, the Specs database was further processed with the PAINS substructure filter developed in our lab using Pipeline Pilot, version 7.5. Then the remaining 182,014 molecules were subjected to moleculardocking to remove low binding affinity compounds or non-binders usingGilde docking software [101] integrated in Maestro 9.0 (Maestro, version 9.0, Schrödinger, LLC, New York, NY, USA, 2009) inXP mode [102] consideringits highest enrichment factor [103] based on our previous tests [92]. Subsequently, top ranking 2000 molecules, with all the previously tested compounds in our previous paper [92] excluded, was subjected to ChemMapper and 2D similarity search. Then 300 compounds of each method were selected, and a total of 520 compounds were obtained after removing the duplicates. In order to cover chemical space with more diverse scaffolds, all the molecules were clustered into 30 groups and finally 44 compounds were selected and purchased from Specs Company (Quezon, Philippines) for biological tests. All the selections were based on the following criteria: (1) In order to get more confident results and more space for future optimization, molecules with simpler structure were chosen; (2) One compound is selected at least in each cluster to get more diverse chemical space; (3) All the molecules with potential reactive functional groups were not our preferences; (4) Molecules with similar structure or dramatic structural differences with DC-S239 were both considered based on our chemical intuitions.

### 2.2. AlphaLISA-Based Biological Tests

**Figure 3:**
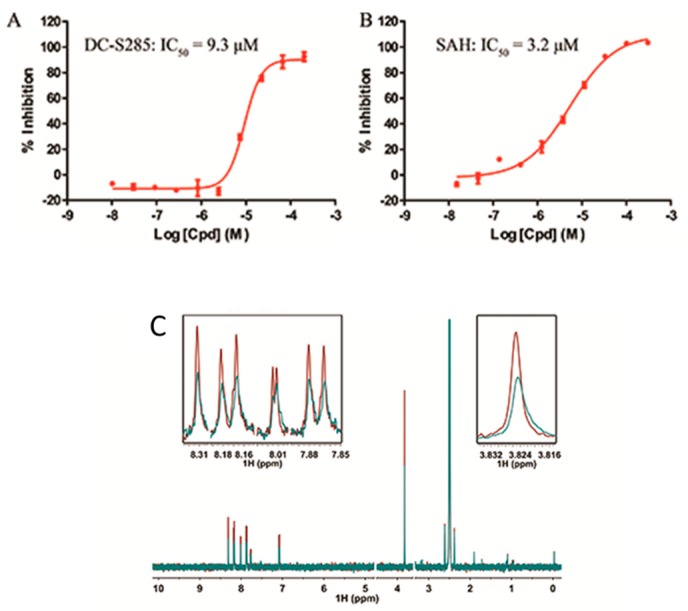
Activity of DC-S285 and of ligand observed 1D NMR experiments. (**A**,**B**). Inhibitory activities of DC-S285 and its reference compound SAH based on the AlphaLISA assay; (**C**). Carr-Purcell Meiboom-Gill Pulse Sequence reveals that DC-S285 binds to SET7. T1ρ spectra acquired by using DC-S285 (colored in red), 20 μM DC-S285 in the presence of 2.5 μM protein (coloured in blue).

All the 44 candidate compounds cherry-picked from similarity search were evaluated for their biochemical activity against SET7 in vitro based on the AlphaLISA assay. SAH was used as the reference compound (Figure 2B). Among them, eight compounds came out at top against SET7 activity with inhibition rate >50% at 100 μM (Figure 1B), resulting in a hit rate of 18%. Notably, compound DC-S285 presented similar potency as the reference compound SAH at the concentration of 100 μM. Then we measured the inhibitory activity at a range of concentrations for DC-S285. Based from dose-response curve, DC-S285 displayed moderate inhibitory activity against SET7 with an IC_50_ value of 9.3 μM while the IC_50_ value of the reference compound SAH was about 3.2 μM (Figure 3A,B).

### 2.3. Validation of DC-S285′s Activity

To rule out the possibility of assay interference of DC-S285, we established the ^3^H-labeled radioisotope methylation assay. The z′ factor value of the established platform is 0.84, demonstrating its robustness for hit validation. DC-S285 and the reference compounds SAH presented the inhibitory activity with the IC_50_ values of 19.5 μM and 5.4 μM, respectively, which was in accordance with the AlphaLisa results.

In order to further confirm that compound DC-S285 binds with SET7, Carr-Purcell-Meiboom-Gill sequence (CPMG) experiments were performed for hit validation Strong binding signals were clearly observed in T1ρ (Figure 3C) and the result indicatedthe mutually exclusive binding of DC-S285 and SET7 catalytic domain.

### 2.4. Cellular Activity of DC-S285

**Figure 4:**
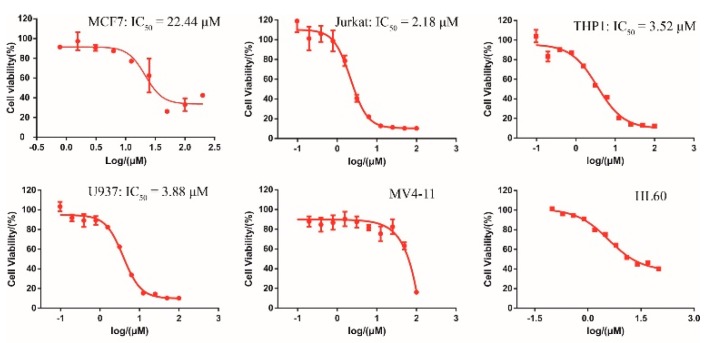
Cellular activity of DC-S285 against different cell lines.

Collectively, both the radioactive and CPMG NMR demonstrated the direct binding between DC-S285 and SET7, wefurther evaluated its antiproliferation activities in several cancer cell lines. As depicted in Figure 4, DC-S285 significantly retard cell proliferation includingMCF7 (breast cancer), and Jurkat, THP1 and U937 leukemia cell lines in a dose-dependent manner at micromolar potencies (Figure 4).

### 2.5. Similarity-Based Analog Searching and SAR Analysis

Based on the biological test, the hit, DC-S285, was used as the promising scaffold for the further structure-activity relationship study (SAR). A second round 2D molecular fingerprint based similarity search was conducted using Pipeline Pilot, version 7.5 to explore the SAR and more potent inhibitors.

**Figure 5:**
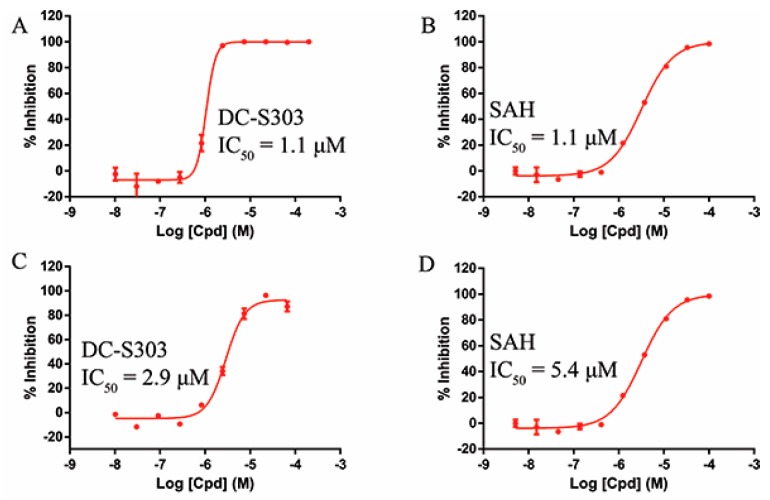
Enzymatic activity of DC-S303 against SET7. (**A**) IC_50_ value of DC-S303 in AlphaLisa assay; (**B**) IC_50_ value of the reference compound SAH in AlphaLisa assay; (**C**) IC_50_ value of DC-S303 in radioactive assay; (**D**) IC_50_ value of the reference compound SAH in radioactive assay.

Some functional groups of DC-S285 were removed intentionally for similarity search, making it possible to explore the structure−activity relationship (SAR) of compound DC-S285 and for further chemical modifications. All the molecules were selected based on cluster analysis and selection criteria described before. The biological activity was measured with AlphaLISA assay at 100 μM and the IC_50_ values of the 10 compounds with inhibition rate over 50% were determined, among which DC-S303 was the most potent SET7 inhibitor with the IC_50_ value of 1.1 μM (Figure 5).

The SAR was summarized in Table 2. It can be concluded that the nitro group in R1 part is essential for activity comparing all the compounds from DC-S303 to DC-S313. If the nitro group is replaced by a chlorine atom, the activity decreases dramatically. What′s more, the *meta* substituent of the nitro group is more favorable (DC-S303 and DC-S304) while DC-S305 displays no activity against SET7. DC-S301 presented moderate inhibitory activity with IC_50_ value of13 μM, indicating the possibility that the benzene ring can be substituted by other aromatic ring with similar size. With the nitro group substituted at the *meta* position and chlorine atom at the *ortho* position, the comparison of compounds DC-S315, DC-S317, DC-S318, DC-S324, DC-S327 indicates that if the aromatic ring is not directly linked with R2 part or there is no aromatic ring linked with R2 part, the activities against SET7 decrease. DC-S314 is an exception possibly because of the flexible alkane chain meaning that it can adapt a suitable conformation to bind with SET7. Moreover, a single aromatic ring with a proper substituent will contribute to better activity. For example, if R3 is the benzene ring or a bromine substituted one, the activity is much higher than other ones (DC-S328 and DC-S333). The furan ring can contribute as an aromatic ring, but less favorable than benzene ring (DC-S329 with IC_50_ value = 92 μM). And it can be concluded that the diphenyl ring is the best candidate for R3 based on compound DC-S303. When R2 and R3 are fixed (from DC-S365 to DC-S364), nitro group at para position with a different R2 group from previous discussions contribute to better activity like DC-S334, but not for other substitution groups in benzene ring or aryl linkers. The rest of this table supports that the linker *A* is the best suitable choice.

### 2.6. Selectivity of DC-S303

A qualified lead compound or chemical probe should feature not only potent binding affinity, but also goodselectivity. Considering that besides SET7, there are some other methyltransferases that share the same cofactor and similar substrate pocket, we further tested the inhibition ratios of DC-S303 against other epigenetic targets, including SETD1B, SETD8, G9a, SMYD2 and EZH2 in vitro (Table 3). The results suggested that this compound displayed moderate selectivity against epigenetic targets that underscored its value for further optimization.

### 2.7. Binding Mode Prediction of DC-S303

**Figure 6:**
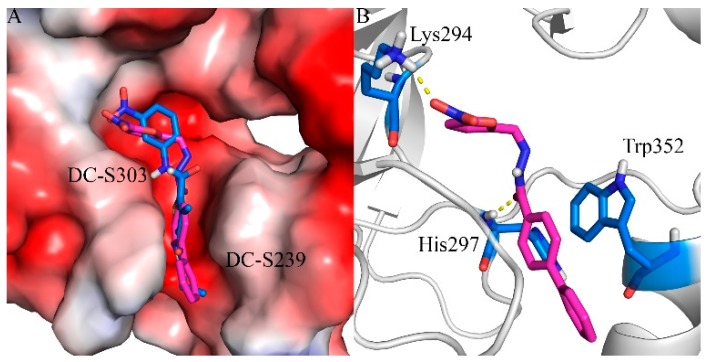
Predicted binding mode of DC-S303 against SET7. (**A**) Binding conformation alignment of DC-S303 and DC-S239. All the compounds are shown in sticks and the protein is shown in surface; (**B**) Putative binging mode of DC-S239 and important polar interactions. The compound is shown in magentas sticks and key residues are displayed in blue sticks.

In order to predict the putative binding mode, a docking calculation was conducted as mentioned before. The proposed binding mode (Figure 6) suggests that it shares similar binding with the previous reported compound DC-S239 at the SAM binding region. It forms a key hydrogen bond with residue Lys294, which is reported to be a potential factor for selective SET7 inhibitor design. The linking benzene of the diphenyl group forms π-π stacking interactions with Trp352, stabilizing its binding into the SAM pocket. The hydrogen bond between DC-S303 and SET7 contributes to the orientation by pulling the middle of this compound.

## 3. Materials and Methods

### 3.1. Virtual Screening: Ligand Database Preparation

The Specs commercial database, containing approximately 200,000 molecules, was filtered using Pipeline Pilot, version 7.5 (Pipeline Pilot; Accelrys Software Inc., San Diego, CA, USA) based on Lipinski′s Rule of Five [96] in order to get promising molecules with good drug-likeproperties. What′s more, the ‘pan-assay interference compounds’ (PAINS) were also removed using the substructure filter protocol developed in our lab with Pipeline Pilot, version 7.5 [97,98,99,100]. As for those molecules for docking calculations, they were subjected to LigPrep to generate all stereoisomers and different protonation states with Epik [104].

### 3.2. Virtual Screening: ProteinPreparation

The protein structure was processed as previously described [92]. Twenty-nine X-ray crystal structures of SET7 are available in the PDB database, and the SET domain of these structures were aligned for comparison and root mean square deviation (RMSD) calculation, which is conserved in the SET domain-containing family. We obtained a maximum root mean-square derivation (RMSD) value of 0.37, suggesting that the three-dimensional structure of the SET domain in SET7 is conserved. Considering the structure resolution and integrity, the crystal structure of SET7 complex with SAM (PDB ID: 1N6A) was chosen for docking. The protein was prepared with Protein Preparation Wizard Workflow, as provided in Maestro, with a pH value of 7.0 ± 2.0. Other parameters were set as the default.

### 3.3. Virtual Screening: 2D Molecular FingerprintBased Similarity Search

2D molecular fingerprint based similarity search was conducted with similarity search protocol implemented in Pipeline Pilot, version 7.5. Top-ranked 300 molecules were selected for further investigation. As for the SAR part of DC-S285, the similarity search is also performed as described in our previous published paper.

### 3.4. Virtual Screening: Scaffold Hopping Based Similarity Search

To get compounds with similar 3D conformations, scaffold hopping was performed with ChemMapper web server (http://lilab.ecust.edu.cn/chemmapper/) [93] against Specs molecule library. Other parameters were set as default.

### 3.5. SET7 Inhibition Assays

AlphaLISA assays were applied to determine the inhibitory activity targeting SET7. The purified SET7 protein was incubated in modified Tris buffer in 384-well plates (Perkin Elmer, Cat. No. 6007299, Waltham, MA, USA) at room temperature for 15 min. The compounds were transferred to the assay plate using Echo in 100% DMSO, and substrate solution was added to each well to start the reaction. Acceptor and donor beads were added and incubated for 60 min at room temperature, shielded from light. The endpoint was evaluated with EndSpire in Alpha mode. The experimental data was fitted in GraphPad Prism 5 to obtain inhibition values using the equation as follows:

(1) $$\mathrm{Inhibition}\;\%=\;\frac{Max-Siginal}{Max-Min}\times 100\%$$

As for ^3^Hradioactive methylation assay, SAH was used as the reference compound and the compounds were tested in 10 concentrations in duplicate in modified Tris buffer (1× assay buffer). The following materials were purchased: SET7 (Enzo, Cat. No. ALX-201-178, New York, NY, USA), [^3^H]-SAM (PerkinElmer Inc., Lot. No. 1790854), SAH (Sigma, Cat. No. A9384-25MG, St. Louis, MO, USA) and 384-well Flashplate (Perkin Elmer, Cat. No. SMP410A001PK,). The compounds were transferred to the assay plate by Echo 550 with 3-fold dilution in 100% DMSO where the final DMSO concentration is 1%. Then the enzyme solution was transferred to the assay plate and 1× assay buffer was transferred for low control. After 15 min incubation at room temperature, peptide and [^3^H]-SAM mix solution was transferred to each well to start the reaction. The cold SAM in 1× buffer was added to each well to stop the reaction after 60 min incubation at room temperature. 25 μL of the whole reaction system per well was transferred to Flashplate which was washed with dH_2_O and 0.1% Tween-20 for three times after 60 min incubation. The data was read on Microbeta. All the inhibition assays are performed in duplicate.

### 3.6. Enzymatic Selectivity Assays

For SETD1B, G9a and SMYD2, the activities against those targets were tested in modified Tris buffer (1× assay buffer). All the solid compounds were dissolved to 20 mM in 100% DMSO. The materials used were SETD1B (Active Motif, Cat. No. M1083, Carlsbad, CA, USA), G9a (BPS, Cat. No. 51001, San Diego, CA, USA), SMYD2 (Active Motif, Cat. No. 31323, c), G9a (BPS, Cat. No. 51001,) and 384-well plate (Perkin Elmer, Cat. No. 6007299). Both the enzyme solution and the substrate solution were prepared in 1× assay buffer enzyme solution or 1× assay buffer for low control was transferred to the assay plate. Then substrate mix solution was added to each well to start the solution for 15 min incubation at room temperature. For SETD1B, G9a, the incubation time was adjusted to 60 min and for SMYD2, the incubation time was set to 240 min. For detection, acceptor and donor beads were added and incubated for 60 min at room temperature, shielded from light. The endpoint was evaluated with EnSpire in Alpha mode.

The radioactive methylation inhibition assay of SETD8 was performed in modified Tris buffer. H3K27me peptide and [^3^H]-SAM (PerkinElmer, Cat No. NET1551MC) were added in 1x buffer as the substrate solution. The enzyme solution was incubated at room temperature for 15 min before substrate solution was added to each well to start the reaction. Cold SAM (Sigma, Cat. No. 7007-100MG) was added in 1x buffer to prepare the stop mix, and was added to stop the reaction; finally 10 μL of the reaction system was transferred to flashplate (PerkinElmer Inc., Cat. No. SMP410A001PK) and incubated at room temperature for a minimum of 2 h. The plate was washed three times with dH_2_O and 0.1% Tween-20, and the radioactivity signal was detected by liquid scintillation counting (MicroBeta, PerkinElmer). SAH was used as the reference compound.

The radioactive methylation inhibition assay of EZH2 was performed in modified Tris buffer. H3K27me peptide and [^3^H]-SAM (Perkin Elmer Inc. Waltham, MA, USA, Lot. No. 1731619) were added in 1x buffer as the substrate solution. The enz the yme solution was incubated at room temperature for 15 min before substrate solution was added to each well to start the reaction. Cold SAM (Sigma, Cat. NO. 7007) was added in 1× buffer to prepare stop mix (final concentration 0.5 mM), and was added to stop the reaction; finally 25 μL of the reaction system was transferred to a FlashPlate (Perkin Elmer, Cat. No. SMP410A001PK) and incubated at room temperature for a minimum of 1 h. The plate was washed three times with dH_2_O and 0.1% Tween-20, and the radioactivity was determined by liquid scintillation counting (MicroBeta). GSK-126 was used as the reference compound.

### 3.7. NMR Experiment

Ligand observed T1ρ NMR experiment were applied to investigate ligand-protein interactions. All NMR spectra were acquired at 25 °C on a 600 MHz Bruker Avance III spectrometer equipped with a cryogenically cooled probe (Bruker Biospin, Ettlingen, Germany). Samples containing 20 μM DC-S285, and 20 μM DC-S285 in the presence of 2.5 μM SET7 were dissolved in Tris-HCl buffer (50 mM Tris-HCl, 100 mM NaCl, pH 7.4, 5% DMSO, 95% D_2_O) and then used in NMR data acquisition. T1ρ spectra were recorded by using the pulse sequence of solvent-suppressed ^1^D ^1^H CPMG (cpmgPr1d). The 90° pulse length was adjusted to about 11.80 μs. A total of four dummy scans and 64 free induction decays (FIDs) were collected into 64 K acquisition points, covering a spectral width of 12 kHz (20 ppm) and giving an acquisition time (ACQ) of 2.73 s. STD data was acquired using 4 dummy scans and a relaxation delay of 3 s, followed by a 40 dB pulsed irradiation at frequency of −1.0 ppm or 33 ppm alternatively. The total acquisition time for STD spectrum was 23 min with 128 FIDs.

### 3.8. Cell Culture and Cell Viability Assay

MCF7, HL60, MV4-11, K562, Kasumi-1, U937, THP1, and Jurkat cell lines were purchased from American Type Culture Collection (Manassas, VA, USA). Fetal bovine serum was purchased from Life Technologies. MCF7 cells were cultured in DMEM medium supplemented with 10% fetal bovine serum at 37 °C in an incubator with 5% CO_2_ atmosphere. HL60 and MV4-11 cells were cultured in 1640 medium supplemented with 10% fetal bovine serum. K562, Kasumi-1 and HL60 were cultured in 96 plate at 10,000/well for 2 h. DC-S285 was dissolved in DMSO (Sigma) and then stored at 4 °C. The cells were incubated with DC-S285 at different concentrations ranging from 0 to 100 µM for approximately 72 h. The activities of DC-S239 against MCF7, HL60, MV4-11, K562, Kasumi-1, U937, THP1, and Jurkat were measured by the alamarBlue assays and MTT assays.

## 4. Conclusions

As a key member of the histone methyltransferase family, SET7 has been reported to play diverse biological roles, including cell proliferation, transcriptional network regulation in embryonic stem cell, cell cycle control, protein stability, heart morphogenesis and development. The dysfunction of SET7 is involved in the pathogenesis of several diseases including alopecia areata, breast cancer, tumor and cancer progression, atherosclerosis in human carotid plaques, chronic renal diseases, diabetes, obesity, ovarian cancer, prostate cancer, hepatocellular carcinoma, and pulmonary fibrosis. Despite its controversial role in multiple diseases, the aberrant expression patterns has been observed in the onset and progression of cancers. In peripheral blood mononuclear cells of patients, the histone modification patterns were altered and the expression of SET7 was elevated [69]. Emerging evidence has also demonstrated its role in solid tumors. Zhang et al. demonstrated that SET7 interacts with transcription factor GATA1 and promotes downstream VEGF transcription and tumor angiogenesis [40]. Inhibition of SET7 activity by the SET7 inhibitor cyproheptadine reduced the estrogen receptor alpha expression in MCF7 cells that is important for cancer progression, phencopying the SET7 knockdown studies [37].

Therefore, there is urgent need to develop novel SET7 inhibitors for further detailed chemical biology investigations as well as drug design for cancer therapy.

Several attempts have been made to develop SET7 inhibitors. Both (*R*)-PFI-2 and cyproheptadine were reported to bind with the peptide binding site while other inhibitors lack potencyor selectivity. Recently, we have reported the first cofactor-competitive SET7 inhibitor, DC-S239, with the help of structure-based drug design methods and chemical modifications. However, the chemotypes of current SET7 inhibitors are limited and no SET7 inhibitors have progressed into clinical trials. Thus, potent, selective SET7 inhibitors with novel scaffolds still need to be developed.

In this paper, the previous identified DC-S239 was used as the starting point for scaffold hopping and 2D fingerprint based similarity search leading to the identification of DC-S285, Both radioactive methylation assays and CPMG assays validate the binding between DC-S285 and SET7. In addition, in cellular studies, DC-S285 could significantly inhibit cancer cell proliferation in a dose-dependent manner with micromolar potencies.

Then in the second round similarity search based on DC-S285, the more potent compound DC-S303 was identified with an IC_50_ value of 1.1 μM. In vitro selectivity profiling demonstrated its moderate selectivity against other methyltransferases. Combined with molecular docking result, we carried out the SAR study that shed light on future medicinal chemistry optimizations. This promising compound will facilitate the SET7 related biology studies and provide a good scaffold for future drug design and development. 

## Figures and Tables

**Table 1:** SET7 substrates and its biological functions.

Substrates		Functions	References
Histone	H3K4	transcriptional activation	[27,28]
	H2A		[29]
	H2BK15		[29]
	H1.4		[30]
Non-histone	ARTD1	stimulating poly-ADP-ribose formation after oxidative stress	[31]
	COL2A1	morphology-dependent COL2A1 gene transactivation	[32]
	DNMT1	protein stability regulation of DNMT1 (destabilization)	[33,34]
	E2F1	regulation of E2F1 stabilization co-activator in response to DNA damage	[35,36]
	ERα	protein stability regulation of ERα (stabilization) and enhancing transcriptional activity	[20,37]
	FoxO3	protein stability regulation of FoxO3 (destabilization)	[38]
	FXR	transcriptional activation of FXR-target genes	[39]
	GATA1	required for GATA1-induced breast tumour angiogenesis and growth in nude mice; poor prognostic factors in breast cancer	[40]
	Gli3	activation of Sonic Hedgehog pathway in mammals	[41]
	HIF-1α	promoting HIF-1α protein stability in hypoxia and enhancing HIF-1 mediated glycolytic gene transcription	[21]
	HIF-1α/2α	negatively regulation HIF-α transcriptional activity and HIF-1-mediated glucose homeostasis	[42,43]
	IFITM3	negatively affected IFITM3 antiviral activity	[44]
	MCP-1	regulation of MCP-1 mRNA expression	[45]
	MYPT1	protein stability regulation of MYPT1 (stabilization)	[23]
	p21		[46]
	p53		
	p65 (RelA)	regulation of NF-κB activity	[11,47,48]
	PCAF		[49]
	PDX1	maintenance of Pdx1 activity and β cell function; control insulin gene expression based on glucose concentration	[50,51,52]
	PCG-1α		[53]
	pRb	cell cycle arrest	[13,14]
	RARα		[54]
	RB	promotes cell cycle progression	[55]
	NFE2L2	negatively regulates the expression of NFE2L2 and its downstream genes	[56]
	Smad7		[57,58]
	SIRT1	inducing the dissociation of SIRT1 from p53 and increasing p52 activity	[59]
	STAT3	negatively regulation of protein stability and transactivation activity	[22]
	SUV39H1	gene instability and cell proliferation inhibition	[12,60]
	TAF7	RNA polymerase ii-dependent transcription coactivator	[61]
	TAF10	RNA polymerase ii-dependent transcription coactivator	[62]
	TAT	enhancing HIV transcription	[63]
	TGF-β1	transcriptional activation of fibrotic genes	[64,65]
	TP2	elongating to condensing spermatids	[66]
	YAP	control YAP subcellular localization and function	[67]
	YY1	regulation of YY1 DNA-binding activity	[68]
	AKA6	unknown	[29]
	CENPC	unknown	[29]
	MeCP2	unknown	[29]
	MINT	unknown	[29]
	PPARBP,	unknown	[29]
	ZDH8	unknown	[29]
	Cullin1	unknown	[29]
	IRF1/2	unknown	[29]

**Table 2:**
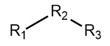
Structure-Activity Relationship (SAR) of DC-S303 and its derivatives.

No.	R_1_	R_2_	R_3_	Inhibition Ratio at 100 μM/%	IC_50_ (μM)
DC-S303	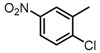	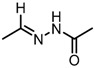 (A)	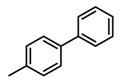	99	1.1
DC-S304	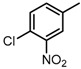			44	
DC-S305	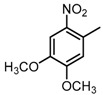			−5	
DC-S306	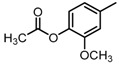			94	20
DC-S307	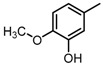			8	
DC-S308	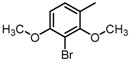			7	
DC-S309	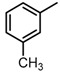			−5	
DC-S310	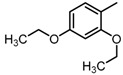			−6	
DC-S311	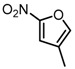			97	13
DC-S312	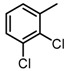			−3	
DC-S313	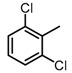			−6	
DC-S314	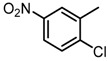	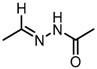 (B)	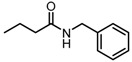	77	46
DC-S315			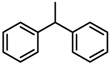	46	
DC-S316			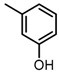	37	
DC-S317			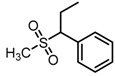	29	
DC-S318			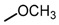	17	
DC-S319			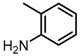	14	
DC-S320			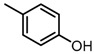	11	
DC-S321			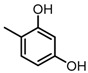	9	
DC-S334	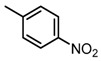	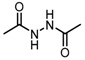 (C)	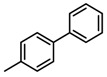	96	9.9
DC-S335	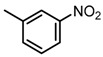			12	**IC_50_ (μM)**
DC-S336	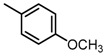			49	1.1
DC-S337	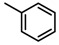			1	
DC-S338	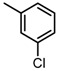			1	
DC-S339	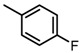			−6	20
DC-S340	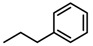			−6	
DC-S341	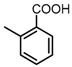			−10	
DC-S342	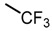			−13	
DC-S343	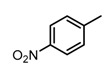	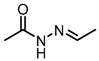 (D)	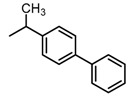	1	
DC-S344			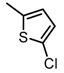	32	13
DC-S345			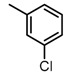	29	
DC-S346			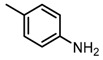	21	
DC-S347			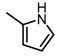	10	
DC-S348			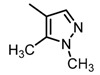	−11	
DC-S349			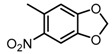	−1	
DC-S350			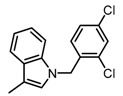	6	
DC-S351			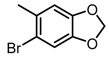	96	3.4
DC-S352			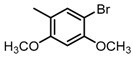	39	
DC-S353			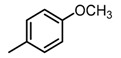	35	
DC-S354			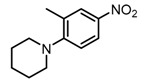	35	
DC-S355			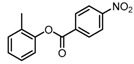	20	
DC-S356			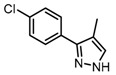	17	
DC-S357			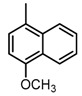	13	
DC-S358			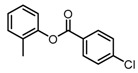	6	
DC-S359			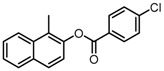	5	
DC-S360			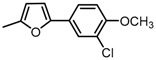	4	
DC-S361			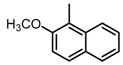	−1	
DC-S362			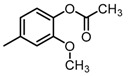	−4	
DC-S363			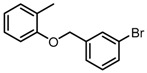	−15	
DC-S364			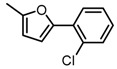	54	3.7
DC-S365	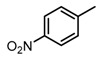	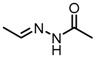 (E)	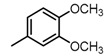	10	
DC-S366			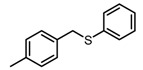	9	
DC-S367			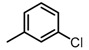	−1	

**Table 3:** Selectivity of DC-S303 over other epigenetic targets.

Compound No.	Target	Inhibition Ratio at 100 μM/%
DC-S303	SETD7	90.51
	SETD1B	27.12
	SETD8	55.23
	G9a	52.56
	SMYD2	24.55
	EZH2	47.88
